# Imaging of α7 nicotinic acetylcholine receptors in brain and cerebral vasculature of juvenile pigs with [^18^F]NS14490

**DOI:** 10.1186/s13550-014-0043-5

**Published:** 2014-08-05

**Authors:** Sven Rötering, Winnie Deuther-Conrad, Paul Cumming, Cornelius K Donat, Matthias Scheunemann, Steffen Fischer, Guoming Xiong, Jörg Steinbach, Dan Peters, Osama Sabri, Jan Bucerius, Peter Brust

**Affiliations:** 1 Institute of Radiopharmaceutical Cancer Research, Helmholtz-Zentrum Dresden-Rossendorf, Permoserstr. 15, Leipzig 04318, Germany; 2 Department of Nuclear Medicine, Friedrich-Alexander-Universität, Ulmenweg 18, Erlangen 91054, Germany; 3 Department of Pharmacology and Neuroscience, Copenhagen University, Blegdamsvej 3B, Copenhagen 2200, Denmark; 4 Department of Nuclear Medicine, Ludwig-Maximilians-Universität, Marchioninistr. 15, Munich 83177, Germany; 5 DanPET AB, Rosenstigen 7, Malmö SE-21619, Sweden; 6 Department of Nuclear Medicine, Universität Leipzig, Liebigstr. 18, Leipzig 04103, Germany; 7 Department of Nuclear Medicine, Maastricht University Medical Center, P. Debeylaan 25, Maastricht 6229, The Netherlands; 8 Cardiovascular Research Institute Maastricht (CARIM), Maastricht University Medical Center, P. Debeylaan 25, Maastricht 6229, The Netherlands; 9 Department of Nuclear Medicine, University Hospital RWTH Aachen, Pauwelstr. 30, Aachen 52074, Germany

**Keywords:** Alpha7 nicotinic acetylcholine receptors, Alzheimer's disease, Blood-brain barrier, Cancer, Diazabicyclononane, Metabolism, PET, Stroke

## Abstract

**Background:**

The α7 nicotinic acetylcholine receptor (nAChR) is an important molecular target in neuropsychiatry and oncology. Development of applicable highly specific radiotracers has been challenging due to comparably low protein expression. To identify novel ligands as candidates for positron emission tomography (PET), a library of diazabicyclononane compounds was screened regarding affinity and specificity towards α7 nAChRs. From these, [^18^F]NS14490 has been shown to yield reliable results in organ distribution studies; however, the radiosynthesis of [^18^F]NS14490 required optimization and automation to obtain the radiotracer in quantities allowing dynamic PET studies in piglets.

**Methods:**

Automated radiosynthesis of [^18^F]NS14490 has been performed by [^18^F]fluorination with the tosylate precursor in the TRACERlab™ FX F-N synthesis module (Waukesha, WI, USA). After optimization, the radiochemical yield of [^18^F]NS14490 was consistently approximately 35%, and the total synthesis time was about 90 min. The radiotracer was prepared with >92% radiochemical purity, and the specific activity at the end of the synthesis was 226 ± 68 GBq μmol^−1^. PET measurements were performed in young pigs to investigate the metabolic stability and cerebral binding of [^18^F]NS14490 without and with administration of the α7 nAChR partial agonist NS6740 in baseline and blocking conditions.

**Results:**

The total distribution volume relative to the metabolite-corrected arterial input was 3.5 to 4.0 mL g^−1^ throughout the telencephalon and was reduced to 2.6 mL g^−1^ in animals treated with NS6740. Assuming complete blockade, this displacement indicated a binding potential (BP_ND_) of approximately 0.5 in the brain of living pigs. In addition, evidence for specific binding in major brain arteries has been obtained.

**Conclusion:**

[^18^F]NS14490 is not only comparable to other preclinically investigated PET radiotracers for imaging of α7 nAChR in brain but also could be a potential PET radiotracer for imaging of α7 nAChR in vulnerable plaques of diseased vessels.

## Background

The homomeric α7 nicotinic acetylcholine receptor (α7 nAChR), together with the heteromeric α4β2 nAChR, belongs to the cysteine-loop family of ligand-gated ion channels and they represent the two most abundant nAChR populations in the brain [1]. The α7 nAChRs are expressed in all cell types present in the brain, including cortical neurons [2], astrocytes [3], microglia [4],[5], oligodendrocyte precursor cells [6] and endothelial cells [7]. They are implicated in inflammatory processes related to neurodegeneration, psychiatric diseases and brain trauma [8],[9]. Furthermore, neuronal and non-neuronal expression of the α7 nAChR has also been found in peripheral organs [6],[10], and the receptors were proposed to be involved in the development of cancer [8],[11]. Recently, studies indicate an important role for α7 nAChRs in cardiac angiogenesis [12] and in modulation of heart rate dynamics during systemic inflammation [13].

Given the wide distribution and diverse functions of the α7 nAChR, quantitative molecular imaging offers the possibility to improve diagnostics in major fields of nuclear medicine such as oncology, neurology and cardiology. As recently reviewed [14],[15], considerable efforts to develop α7 radioligands suitable for positron emission tomography (PET) imaging have been made in the last decade. This goal is challenging due to the comparably low protein expression under physiological conditions. By use of [^11^C]CHIBA-1001, the only α7-specific PET radiotracer tested in humans so far, only small regional binding differences of uncertain pharmacological specificity were detectable in the brain [16]. Furthermore, the regional distribution of [^11^C]CHIBA-1001 did not match that of the highly selective α7 nAChR radiotracer [^125^I]α-bungarotoxin in monkey and human brain samples [17] which corresponds to the low affinity and selectivity of [^3^H]CHIBA-1001 in rodent brain [18]. Very recently, [^18^F]ASEM has shown promising properties for the quantitative imaging of α7 nAChR in the brain of non-human primates [19]. Up to that, the 1,4-diazabicyclo[3.2.2]nonane derivatives [^11^C]NS14492 [20], [^18^F]NS10743 [21] and [^18^F]NS14490 [22], all based on compounds developed by NeuroSearch A/S (Ballerup, Denmark) [23],[24], were the most qualified promising radiotracers for imaging of α7 nAChR by PET. Of these, [^11^C]NS14492 and [^18^F]NS10743 were assessed by preclinical PET studies in pigs [20],[25]. Although the general suitability has been shown, the magnitude of the binding potential (BP_ND_; approximately 0.5) was at the lower limit for an applicable PET radiotracer. Binding of the higher affine [^18^F]NS14490 [22] in mouse brain slices correlated with the pattern of α7 nAChR expression and was displaced with the α7 nAChR-specific alkaloid methyllycaconitine [8]. Furthermore, brain uptake and target specificity were proven by organ distribution studies in mice [22]. Therefore, to investigate if higher affinity of [^18^F]NS14490 *in vitro* translates into higher binding potential *in vivo*, the radiosynthesis of [^18^F]NS14490 was optimized and automated to obtain the radiotracer in quantities allowing dynamic PET studies in piglets. Furthermore, to prove the hypothesis that [^18^F]NS14490 specifically binds to α7 nAChR expressed in the brain and cerebral vasculature of piglets, blocking studies by administration of the α7 nAChR partial agonist NS6740 were performed.

## Methods

### Animals and drugs

Animal experimental procedures were approved by the Animal Care and Use Committee of Saxony (TVV 22/10). Six female German Landrace pigs (mean weight 15.8 ± 0.8 kg, mean age 8 weeks) were used in this study. Anaesthesia and surgery of the animals were performed as described previously [26].

Reagents and solvents of highest available quality were obtained from Merck KGaA (Darmstadt, Germany), Sigma-Aldrich Co. LLC. (Taufkirchen, Germany), Carl Roth GmbH + Co KG (Karlsruhe, Germany), VWR International GmbH (Darmstadt, Germany) and Fischer Scientific GmbH (Schwerte, Germany). Chemicals and solvents were used without further purification. The reference compound NS14490 (1-(2-fluoroethyl)-6-(5-(1,4-diazabicyclo[3.2.2]nonan-4-yl)-1,3,4-oxadiazol-2-yl)indole) and the blocking compound NS6740 (1,4-diazabicyclo[3.2.2]nonan-4-yl(5-(3-(trifluoromethyl)phenyl)furan-2-yl)methanone; also known as A-793394) was obtained from DanPET AB (Malmö, Sweden) [27].

### Radiochemistry and analytics

No-carrier-added [^18^F]fluoride was obtained from a cyclotron Cyclone®18/9 (iba International, Louvain-la-Neuve, Belgium) by irradiation of [^18^O]H_2_O in a Nirta® [^18^F]fluoride L target with a fixed energy proton beam (18 MeV). [^18^O]H_2_O (Hyox 18 enriched water) was purchased from Rotem Industries Ltd. (Arava, Israel)/Rotem GmbH (Leipzig, Germany) or [^18^O]H_2_O recycled by the in-house established method [28] was used. Radiosyntheses were performed on a TRACERlab™ FX F-N synthesizer from GE Healthcare (Waukesha, WI, USA) equipped with PU-1580 pump from Jasco International Co. (Gross-Umstadt, Germany), a WellChrom K-2001 UV detector from KNAUER GmbH (Berlin, Germany, a NaI(Tl)-counter and an automated data acquisition tool (NINA software version 4.8 rev. 4, Nuclear Interface GmbH, Dortmund, Germany).

Radio-thin layer chromatography (radio-TLC) analyses were accomplished with Polygram® ALOX N/UV254 plates (Macherey-Nagel, Düren, Germany) using methylene chloride/methanol 95:5 (radiosynthesis) and 90:10 (metabolite analysis). The reference spots were visualized with UV (254 and 366 nm). The radio-TLC plates were analysed with a phosphor imager BAS-1800II (Fuji Film, Tokyo, Japan) and evaluated with AIDA 2.31 software (raytest Isotopenmessgeräte GmbH, Straubenhardt, Germany). Labelling yields were calculated as percentages of the product peak relative to the total activity detected.

For analytical radio-high performance liquid chromatography (radio-HPLC), two devices from Jasco International Co. were used (pumps: PU-2080Plus, quaternary gradient: LG-2080-045 and ternary gradient LG-2080-02S, 4-line degasser DG-2080-54 and 3-line degasser DG-2080-53, autosamplers AD-2055Plus, UV/vis detectors UV-2075Plus, coupled with radioactivity flow monitors for HPLC Gabi Star (raytest Isotopenmessgeräte GmbH)). The columns used for analytical radio-HPLC were Multospher® 120 RP18-AQ 250 mm × 4.6 mm, 5 μm (CS-Chromatographie Service GmbH, Langerwehe, Germany), one device equipped with precolumn Multospher® 120 RP18-AQ 40 mm × 4.6 mm, 5 μm (CS-Chromatographie Service GmbH). We used a previously calibrated Wallac 1480 WIZARD 3″ system (PerkinElmer LAS GmbH, Rodgau, Germany) for γ-quantification; radio-HPLC data were corrected for decay and background.

Semi-preparative separations were performed with the following reverse phase columns: Multospher® 120 RP-18 AQ, 5 μm (CS-Chromatographie Service); Reprosil-Pur® C18-AQ, 7 μm; and Reprosil-Gold® 120 C18, 10 μm (Dr. Maisch HPLC GmbH, Ammerbuch-Entringen, Germany), 50 mm × 10 mm (precolumn) and 150 mm × 10 mm. Mobile phase flow rates and compositions are described in captions of the relevant figures. Chromafix® 30 PS-HCO_3_^−^ solid-phase extraction (SPE) cartridges (Macherey-Nagel GmbH) were used for the separation of [^18^F]fluoride from irradiated water, and Chromafix® HR-X, 85 μm (polystyrene/divinylbenzene) cartridges conditioned with 5 mL desorption agent and distilled water were used for product purification after semi-preparative HPLC.

The tosylate precursor 1 was synthesized as described recently [22]. Automated radiosynthesis of [^18^F]NS14490 has been developed, and *in vitro* autoradiography of piglet brain has been performed as described in detail in Additional file 1.

### PET measurement

The PET scans were run in six female juvenile pigs under isoflurane anaesthesia using [^18^F]NS14490. Anaesthesia and surgery of the animals were performed as described previously [26]. In brief, the animals were premedicated with midazolam (1 mg kg^−1^ i.m.) followed by induction of anaesthesia with 3% isoflurane in 70% N_2_O/30% O_2_. All incision sites were infiltrated with 1% lidocaine, and anaesthesia was maintained throughout the surgical procedure with 1.5% isoflurane. A central venous catheter was introduced through the left external jugular vein and used for the administration of the radiotracer and drugs, and for volume substitution with heparinized (50 IE mL^−1^) lactated Ringer's solution (2 mL kg^−1^ h^−1^). An endotracheal tube was inserted by tracheotomy for artificial ventilation (Servo Ventilator 900C, Siemens-Elema, Solna, Sweden) after immobilization with pancuronium bromide (0.2 mg kg^−1^ h^−1^). The artificial ventilation was adjusted to maintain normoxia and normocapnia (Radiometer ABL 500, Copenhagen, Denmark). Polyurethane catheters (Ø 0.5 mm) were advanced through the left and the right femoral arteries into the abdominal aorta to withdraw arterial blood samples for regular monitoring of blood gases and for radiotracer input function measurements. Body temperature was monitored by a rectal temperature probe and maintained at approximately 38°C by a heating pad. After completion of surgery, anaesthesia was maintained with 0.5% isoflurane in 70% N_2_O/30% O_2_, and the animals were allowed to stabilize for 1 hour before PET measurement.

PET imaging was performed according to the protocol described recently [25]. Briefly, the animals were scanned in prone position with the head held in the aperture of a clinical tomograph (ECAT EXACT HR+, CTI/Siemens, Knoxville, TN, USA) using a custom-made head holder. For attenuation and scatter correction, transmission scans were acquired using three rotating ^68^Ge/^68^Ga rod sources. [^18^F]NS14490 (520 ± 150 MBq; *S*_A_: 110 ± 47 GBq μmol^–1^) was applied in 10 mL saline as a 2-min i.v. infusion using by a syringe pump, followed by flushing with 10 mL heparinized saline (50 IE mL^−1^). The emission scan started upon the initiation of the injection, and dynamic emission data were acquired for a total of 240 min. Three animals were investigated under baseline conditions, and another three under blocking conditions with administration of 3 mg kg^−1^ i.v. of the α7 nAChR partial agonist/functional antagonist NS6740 (DanPET AB) at 3 min before radiotracer injection. A continuous infusion of 1 mg kg^−1^ h^−1^ NS6740 was applied throughout the scan.

Arterial blood was sampled continuously using a peristaltic pump during the first 20 min of the recording, followed by manual sampling at 25, 30, 40, 50, 60, 90, and 120 min after injection. After centrifugation, the total plasma radioactivity concentration was measured using a γ-counter (1470 Wizard, PerkinElmer, Shelton, CT, USA) cross-calibrated to the PET scanner. Additionally, arterial whole blood was sampled manually at 4, 16, 30, 60, and 120 min p.i. and plasma obtained as described above to determine the fractions of non-metabolized [^18^F]NS14490. The samples were centrifuged at 13,000 rpm for 2 min to obtain plasma. Eight aliquots of 125 μL from each plasma sample were added to 500 μL ice-cooled acetonitrile, shaken for 2 min, incubated on ice for 10 min and centrifuged at 5,000 rpm for 2 min. The supernatants of each sample series were combined and concentrated to approximately 100 μL for radio-TLC and radio-HPLC analyses as described above. The extraction efficiency was monitored relative to the total radioactivity in aliquots. Bi-exponential fitting of the percentages of untransformed parent in extracts was performed and used to correct the input function of [^18^F]NS14490 for the presence of metabolites.

### Analysis of PET data

After correction for attenuation, scatter, decay and scanner-specific dead time, the images were reconstructed by filtered back projection using a 4.9-mm FWHM Hanning filter into 40 frames of increasing length. A summed PET image of the entire 240-min recording was calculated for each animal and used for alignment with a T1-weighed MR image of a 6-week-old farm-bred pig as described previously [29]. Dynamic time-activity curves (TACs) were calculated for the following volumes of interest (VOIs): frontal cortex, temporal lobe, parietal lobe, occipital lobe, hippocampus, striatum (defined as mean radioactivity in caudate and putamen), cerebellum, thalamus, middle cortex, ventral cortex, midbrain, pons, colliculi, left carotid artery, and circle of Willis. The radioactivity in all VOIs was calculated as the mean radioactivity concentration (Bq mL^–1^) for the left and right sides. To generate standardized uptake values (SUVs in g mL^–1^) the VOI TACs (in kBq mL^–1^) were normalized to the injected dose and corrected for animal weight (in kBq g^–1^).

To investigate the selectivity of α7 nAChR blockade in the cerebral vessels, retrospective calculation of SUV in the left carotid artery and circle of Willis was done using data from a similar study performed with the α4β2-specific radioligand 2-[^18^F]FA-85380 [26].

The kinetics of [^18^F]NS14490 in the brain was calculated for each 240-min TAC relative to the metabolite-corrected arterial input using a Matlab-based in-house program for a one-compartment model with the blood volume fixed at 0.05 mL g^−1^. We thus obtained estimates of the unidirectional blood-brain clearance (*K*_1_; mL g^−1^ min^−1^) and the apparent washout rate from the brain (*k*_*2*_″; min^−1^), and their ratio, defined as the distribution volume (*V*_T_″; mL cm^−3^) and theoretically identical to *V*_T_ = *K*_1_/*k*_2_(1 + *k*_3_/*k*_4_) [30]. The regional binding potential (BP_ND_ *=* (*V*_T_″_region_ − *V*_T_″_blocked_)/*V*_T_″_blocked_) in the unblocked condition was calculated for each brain region relative to the hippocampus in the blocked condition, assumed to be a global surrogate for *K*_1_/*k*_2_, in the absence of specific binding. For cerebral vessels, the individually blocked condition was used to calculate the BP_ND_. *V*_T_″ was also calculated by the method of Logan et al. [31] for the unblocked and blocked conditions using the interval 30 and 120 min for the linearization. The significance of reductions under blockade was assessed by Student's *t* test (one-tailed, unpaired).

Parametric maps of distribution volume *V*_T_″ were calculated for each animal investigated under baseline and blocking conditions using the metabolite and free fraction corrected arterial input function and the Logan reference tissue method as implemented in PMOD version 3.5 (PMOD technologies, Ltd., Zurich, Switzerland) as described previously [25].

## Results and discussion

The current study proposes that the new PET radioligand [^18^F]NS14490 is suitable to specifically image α7 nAChR in the brain and cerebral vasculature of pigs.

This suggestion is derived from the following observations as specified below: the radioligand shows a distribution pattern in piglet brain which is characteristic for α7 nAChR, a highly significant correlation between specific binding *in vitro* and *in vivo*, a sufficient brain extraction (approximately 30%), an SUV which can be significantly decreased by the specific α7 nAChR ligand NS6740 by 24% to 38%, and a NS6740-induced increase in the clearance rate constant *k*_2_″ estimated by one-tissue-compartment modelling of approximately 30% which is consistent with blockade of a specific binding component.

### Automated radiosynthesis

**Figure 1:**
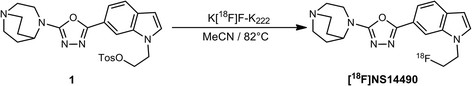
**Radiosynthesis of [**^**18**^**F]NS14490.**

The biological data presented below were obtained based on manual and automated radiosynthesis of [^18^F]NS14490. The manual radiosynthesis of [^18^F]NS14490 was described recently [22] and is based on the development of the automated radiosynthesis for [^18^F]fluspidine [32]. The manual synthesis started with 2.5 to 3 GBq, whereas activities as high as 15 GBq were used for the automated radiosynthesis of [^18^F]NS14490. The procedure included the separation of [^18^F]fluoride from the irradiated [^18^O]H_2_O, the azeotropic drying with Kryptofix K_222_, the radiofluorination of precursor 1 (see Figure 1), semi-preparative HPLC, SPE for purification and solvent exchange, as well as manual evaporation and final formulation of the radiotracer in PBS containing 5% ethanol. The setup of vials and containers of the automatic module and a table showing which vials were used and how they were filled, and which cartridges were used are presented in Additional file 1.

**Figure 2:**
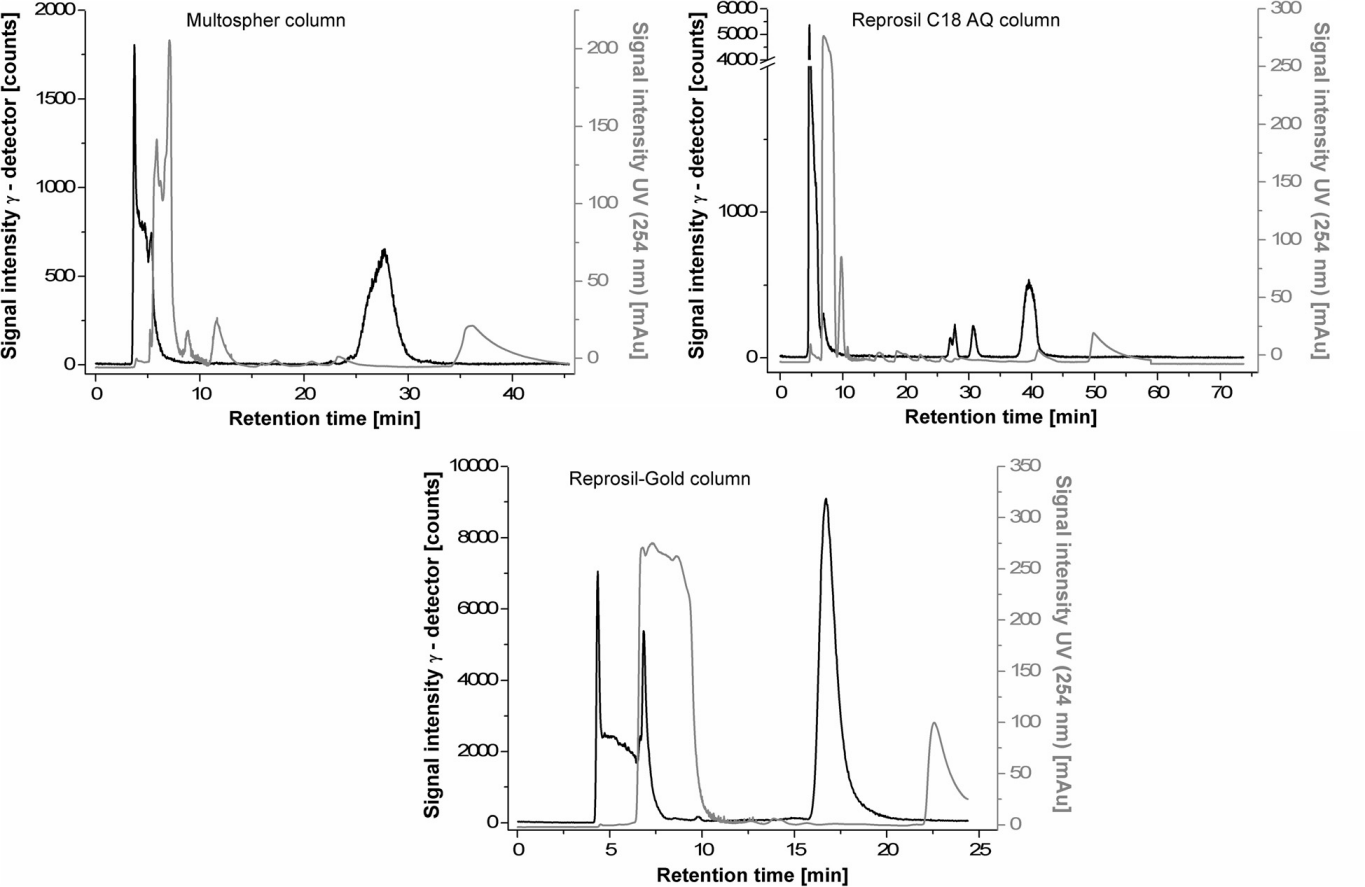
**Semi-preparative RP-HPLC for the isolation of [**^**18**^**F]NS14490 in a module system with different columns.** Multospher column (left; 35% MeCN, 65% water, 12.5 mM ammonium acetate (NH_4_OAc), flow rate 2.5 mL min^−1^), a Reprosil-Pur C_18_ AQ column (right; 35% MeCN, 65% water, 12.5 mM NH_4_OAc, flow rate 2 to 3 mL min^−1^) and a Reprosil-Gold column (bottom; 35% MeCN, 65% water, 20 mM NH_4_OAc, flow rate 2 mL min^−1^).

Initially, semi-preparative HPLC and SPE were optimized as described in the following: Figure 2 (left) shows the semi-preparative isolation of the radiotracer on a Multospher AQ column, as in the manual synthesis [22]. The peak width of the product (*t*_*r*_ = 28 min) is rather broad with the product fraction eluting for more than 5 min. Testing of a Reprosil AQ column shows a retention time of 40 min, despite a flow gradient from 2 to 3 mL min^−1^ (Figure 2 right). The corresponding chromatogram indicates co-elution of product with UV-active substances. Separation with the Reprosil-Gold (Figure 2 bottom) shows a rapid separation of the product (*t*_*r*_ = 17 min) with a narrow peak width, and eventually, this column was utilized in further radiosyntheses.

As previously mentioned for the manual synthesis [22], acidic solvents are necessary for a sufficient desorption of the radiotracer. HR-X cartridges were chosen also for the automated radiosynthesis because their stationary phase provides better pH stability. Unfortunately, ethanol containing 2% acetic acid gave a low elution rate for [^18^F]NS14490 (<50%) unless excessive volume was employed. Elution with acetonitrile containing 1.5% formic acid was nearly quantitative, with 4% ± 3% (*n* = 7) of activity remaining on the cartridges.

After optimisation, the labelling yield of the automated radiosynthesis of [^18^F]NS14490 as determined with radio-TLC was 73% ± 9% (*n* = 7), and the specific activity was 226 ± 68 GBq μmol^−1^ (corrected to the end of synthesis, *n* = 6). The measured radiochemical purity of the product (≥92%) was lower than that previously achieved after manual synthesis (≥95%) [22]. This may be related to (I) the acid sensitivity of the radiotracer as discussed previously [22], (II) the acidic solvent needed for a quantitative elution from the cartridge and (III) the higher volume (1.5 mL) of elution agent needed compared to the manual synthesis [22]. Apparently, the longer evaporation time of the larger volume of the acidic solvent promoted the degradation as the radiotracer was exposed longer to the acidic milieu.

Before using in PET studies of pigs, the suitability of the radiotracer was proven by *in vitro* autoradiography. Brain slices from pigs were incubated with [^18^F]NS14490 and selected α7 nAChR inhibitors. The specific binding estimated by *in vitro* autoradiography showed a highly significant correlation (see Additional file 1) to the PET estimate of specific binding (see below).

### Plasma input function

**Figure 3:**
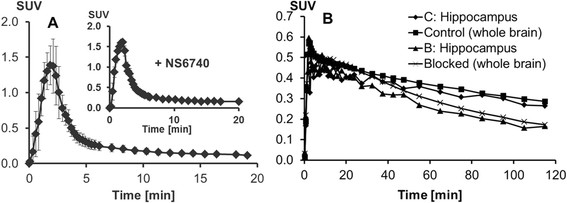
**Time-activity curves obtained during PET experiments. (A)** The metabolite-corrected plasma samples. **(B)** Those from measurement of the whole brain and hippocampus of individual piglets. Experiments were performed under baseline (*n* = 4) and blocking conditions (*n* = 3, inset). Values are means ± S.D.

**Figure 4:**
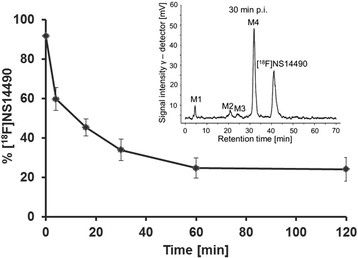
**Percentage of untransformed [**^**18**^**F]NS14490 in plasma as a function of circulation time.** The inset shows a representative chromatogram of a plasma extract at 30 min p.i. The retention time of [^18^F]NS14490 was 41 min. A total of four radiometabolites were detected (M1 to M4).

[^18^F]NS14490 was used in dynamic PET studies in juvenile pigs. The plasma input functions under baseline and blocking conditions are shown in Figure 3A. They were corrected for the presence of radiolabelled metabolites investigated in plasma samples taken at 4, 16, 30, 60 and 120 min p.i. On average, 89% ± 3% of the radioactivity was extracted by plasma protein precipitation. Chromatographic analysis showed a spectrum of four radiometabolites (Figure 4, inset), all of them more hydrophilic than [^18^F]NS14490. The absence of significant cranial uptake suggests that [^18^F]NS14490 is not defluorinated in the pig.

Under control conditions, [^18^F]NS14490 represented 39% and 33% of the total plasma radioactivity at 30 and 60 min p.i. (Figure 4), respectively; pre-treatment with NS6740 was without significant effect on determined metabolite fractions values. Therefore, bi-exponential fitting of the percentages of untransformed [^18^F]NS14490 of all investigated animals was performed (Figure 4), and the obtained empiric function was used to correct the input function of [^18^F]NS14490 for the presence of metabolites.

Comparison of the corrected input functions under baseline and blocking conditions (Figure 3A) shows a 15% to 20% increase during the first minutes after initiation of the NS6740 infusion (baseline = 1.32 ± 0.38 SUV, blocking = 1.60 ± 0.02 SUV at 2 min p.i.).

### Dynamic PET data

The uptake of [^18^F]NS14490 in piglet brain was investigated by PET. The initial tracer influx to the brain attained an SUV_max_ of 0.54 ± 0.07 under the control and of 0.60 ± 0.09 under blocking conditions within the first minutes after injection (Figure 3B). Examples of time-activity curves in the hippocampus and the whole brain for a control animal and an animal treated with a blocking dose of NS6740 are shown in Figure 3B. At the end of the study (between 210 and 240 min p.i.), blocking by NS6740 reduced the SUV by 38% (hippocampus) and 24% (midbrain) in comparison to the controls (*p* < 0.05 in all regions except the midbrain).

Compartmental analysis indicated unidirectional clearance (*K*_1_) of approximately 0.15 mL g^−1^ min^−1^ throughout the brain (Table 1) which suggests an extraction fraction of approximately 30% for the untransformed tracer in the blood, relative to the cerebral blood flow in anaesthetized piglets [33]. The washout was likewise rapid in the untreated piglets, with an apparent *k*_2_″ close to 0.05 min^−1^; the total distribution volume (*V*_T_″) is thus approximately 3.5 mL g^−1^ throughout the brain. The administration of NS6740 did not change the magnitude of *K*_1_. However, *k*_2_″ was increased significantly by NS6740 administration in most brain regions, propagating to a non-significant reduction in the magnitude of *V*_T_″ by 25% to 50% (Table 1); the increase in *k*_2_″ is consistent with blockade of a specific binding component, which however could not be examined further, as a two-compartment reversible binding model did not give stable parameter estimates (data not shown).

**Table 1:** **Kinetic parameters of the blood-brain partitioning of [**^**18**^**F]NS14490 in the brain and brain vasculature**

	**Control (*n*** **= 4)**^**a**^				**Blocked (*n*** **= 3)**^**a**^		
	***K***_**1**_	***k***_**2**_**″**	***V***_**T**_**″**	**BP**_**ND**_	***K***_**1**_	***k***_**2**_**″**	***V***_**T**_**″**
Frontal cortex	0.161 (0.055)	0.046 (0.003)	3.78 (1.01)	0.45	0.163 (0.029)	0.059 (0.003)*	2.82 (0.62)
Temporal cortex	0.145 (0.034)	0.040 (0.003)	3.98 (1.02)	0.53	0.145 (0.028)	0.049 (0.001)**	3.04 (0.69)
Parietal cortex	0.149 (0.037)	0.044 (0.001)	3.63 (0.86)	0.40	0.155 (0.034)	0.056 (0.004)*	2.81 (0.61)
Occipital cortex	0.171 (0.064)	0.051 (0.008)	3.59 (0.84)	0.40	0.164 (0.045)	0.062 (0.005)***	2.70 (0.59)
Basal forebrain	0.163 (0.035)	0.048 (0.006)	3.75 (0.74)	0.44	0.164 (0.040)	0.055 (0.002) n.s.	3.04 (0.72)
Hippocampus	0.146 (0.044)	0.045 (0.005)	3.50 (0.68)	0.35	0.154 (0.035)	0.062 (0.010)***	2.60 (0.61)
Striatum	0.137 (0.029)	0.043 (0.004)	3.48 (0.80)	0.34	0.149 (0.038)	0.055 (0.001)**	2.76 (0.71)
Thalamus	0.160 (0.032)	0.049 (0.003)	3.54 (0.69)	0.35	0.177 (0.039)	0.065 (0.001)*	2.78 (0.68)
Midbrain tegmentum	0.161 (0.026)	0.053 (0.007)	3.47 (0.99)	0.33	0.159 (0.034)	0.062 (0.004) n.s.	2.59 (0.61)
Colliculi	0.162 (0.035)	0.059 (0.003)	3.05 (0.71)	0.17	0.178 (0.042)	0.071 (0.006)**	2.53 (0.50)
Pons	0.205 (0.090)	0.066 (0.010)	3.33 (0.85)	0.28	0.201 (0.034)	0.077 (0.011) n.s.	2.72 (0.84)
Cerebellum	0.186 (0.078)	0.061 (0.005)	3.15 (0.96)	0.21	0.193 (0.064)	0.070 (0.003)***	2.80 (0.96)
Full brain	0.170 (0.054)	0.050 (0.004)	3.56 (0.85)	0.37	0.166 (0.032)	0.059 (0.001)**	2.78 (0.66)
Carotid artery	0.406 (0.194)	0.059 (0.015)	6.71 (1.37)	0.18	0.367 (0.095)	0.064 (0.002) n.s.	5.71 (1.37)
Circle of Willis	0.285 (0.136)	0.050 (0.012)	5.37 (1.35)	0.11	0.254 (0.132)	0.051 (0.021) n.s.	4.83 (0.86)

Data are obtained from a one-tissue compartment model. Values are means ± S.D.; n.s., not significant. ^a^The unidirectional blood-brain clearance (*K*_1_; mL g^−1^ min^−1^), the apparent washout rate constant from the brain (*k*_2_″; min^−1^) and the total distribution volume (*V*_T_″; mL g^−1^) were calculated with plasma volume fixed at 0.025 mL g^−1^. In the unblocked condition, we calculated the binding potential BP_ND_ relative to *V*_T_″ in the blocked condition. Significant difference between *k*_2_″ in the two groups: *p < 0.001, **p < 0.005, ***p < 0.01.

We used the lowest telencephalic magnitude of *V*_T_″ estimated under blocking conditions (hippocampus; 2.6 mL g^−1^) as a global surrogate for *V*_T_″, i.e. the ratio *K*_1_/*k*_2_, of [^18^F]NS14490 in the absence of specific binding. Relative to this, the apparent BP_ND_ ranges from moderate 0.2 in the cerebellum and midbrain to a maximum of nearly 0.5 in the cerebral cortex (Table 1).

Furthermore, we observe evidence for a specific α7 nAChR binding component in the large arteries supplying the brain. The SUV of [^18^F]NS14490 at the end of the study was 36% and 24% reduced by treatment with NS6740 in the carotid artery (*p* < 0.05) and in the circle of Willis, respectively. Retrospective analysis of a similar study performed with the α4β2-specific radioligand 2-[^18^F]FA-85380 [26] did not reveal any alterations of SUV under receptor blockade (control 0.604 ± 0.240, blocked 0.604 ± 0.124).

**Figure 5:**
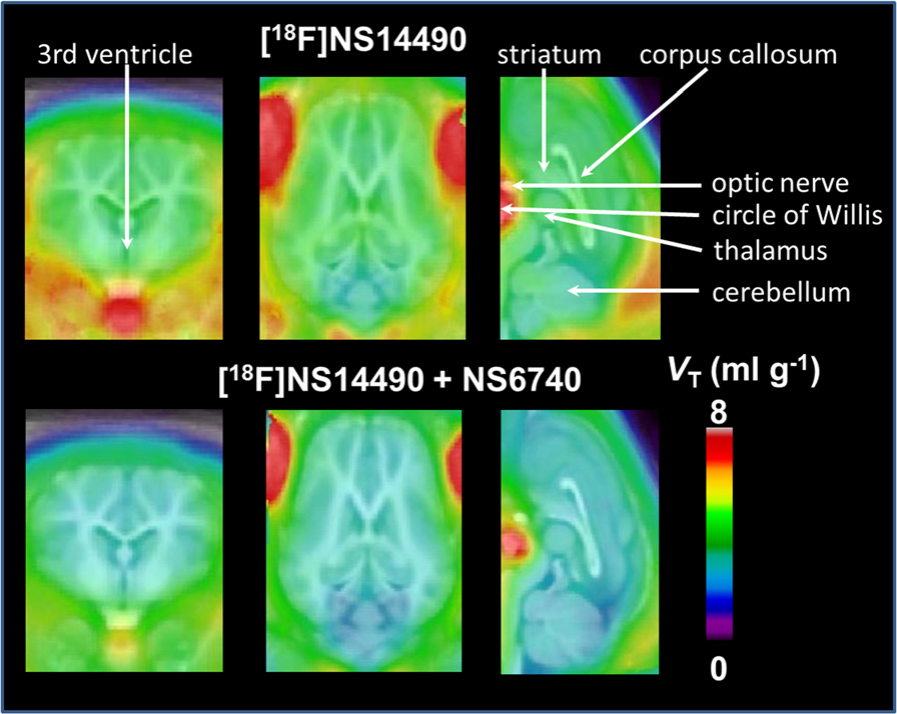
**Parametric maps of the distribution volume (*V***_**T**_**) of [**^**18**^**F]NS14490 in the brain of piglets.** Experiments were carried out under control (unblocked) conditions and with pre-treatment with the competitor NS6740 (blocked). Parametric maps are projected onto an MR atlas for the pig brain and represent the mean of (*n* = 4) control animals and (*n* = 3) animals with blocking. Data are given in mL g^−1^.

The effect of α7 nAChR blockade with NS6740 on the uptake of [^18^F]NS14490 in the brain and brain vessels is clearly seen on the parametric images of *V*_T_″ calculated by Logan analysis (Figure 5). The *V*_T_″ values from the Logan plots are highly correlated (*r* = 0.99) to the *V*_T_″ values from the compartmental analysis (see Additional file 1).

Several lines of evidence show that α3, α4, α5, α7, α10, β2, β3 and β4 nAChR subunits are expressed on human vascular endothelial and smooth muscle cells and that they mediate some of the deleterious effects of nicotine in the vessels [34],[35]. The homogeneous α7 nAChRs primarily mediate endothelial cell proliferation, invasion and angiogenesis. Nicotine induces the expression of endothelial growth factors such as bFGF, PDGF-BB, TGF-α and VEGF in the endothelial cells explaining the pro-angiogenic nAChR-mediated effect of nicotine. These growth factors are also overexpressed in atherosclerotic plaques [35],[36]. Nicotine promotes atherosclerotic plaque neovascularization and may enhance plaque vulnerability, since this critically depends on neovascularization and changes in endothelial connexin expression [37]. Vascular smooth muscle cells particularly express α3, α4, α5, α7 and α10 nAChR subunits. They contribute to angiogenesis and the control of smooth muscle cell proliferation [34]. Interestingly, some of the named nicotine-related vascular effects were even found at concentrations equivalent to plasma levels observed in only moderate smokers [36].

At the time of first publication, [^18^F]NS14490 showed so far the highest α7 nAChR affinity (2.5 nM) among the published 1,4-diazabicyclo[3.2.2]nonane derivatives in addition with 40- and 400-fold selectivity towards human α3β4 and α4β2 nAChR, respectively [14],[22]. Accordingly, an automated radiosynthesis has been developed for further preclinical PET studies in pigs. After the described optimisation, the labelling yield of the automated radiosynthesis (73% ± 9%; *n* = 7) as determined with radio-TLC was identical to that of the manual synthesis (71% ± 9%; *n* = 12) [22]. The radiochemical yield (35% ± 4%) was comparable to that for the previously published radiochemical automatic synthesis of [^18^F]fluspidine [32] the module parameters of which were used as template for the current procedure.

Regarding metabolic stability, species-specific differences were observed with a faster metabolism of [^18^F]NS14490 in piglets than in mice (at 60 min p.i. [^18^F]NS14490 accounts for 33% and 56% of plasma activity, respectively) [22]. In pig plasma, chromatographic analysis showed a spectrum of radiometabolites similar to that seen in mouse plasma. Since metabolite M4 (Figure 4), eluting just prior to the parent compound and detected at comparable levels in both mouse and pig, was nearly absent in mouse brain [22], it is unlikely that M4 penetrates into the pig brain in the present study.

Very recently, the novel 1,4-diazabicyclo[3.2.2]nonane derivative [^18^F]ASEM has been reported possessing an eightfold higher affinity than NS14490 [19]. Accordingly, the distribution volumes *V*_T_ of [^18^F]ASEM in monkey brain were about sixfold higher than the *V*_T_ values of [^18^F]NS14490 in the pig brain as calculated in this study. Differently from our study, the α7 nAChR-selective partial agonist SSR180711 [38] has been used for displacement, and administration of 5 mg kg^–1^ blocked about 80% of [^18^F]ASEM binding in baboon brain [19].

Using NS6740 as α7 nAChR-selective inhibitor, we observed a decrease of *V*_T_ of [^18^F]NS14490 in pigs which was caused by an increase in *k*_2_″. This result indicates NS6740-mediated blockade of a specific binding component. However, this could not be examined further, as a two-compartment reversible binding model did not give stable parameter estimates. An alternative explanation for the observed increase of *k*_2_″ could be an increased washout of tracer due to cerebral blood flow effects of NS6740 as was reported for the PET imaging of D_3_ receptors under antagonistic blockade [39]. However, this is unlikely to be an issue in the herein investigated NS6740-[^18^F]NS14490 approach, since the washout rate was differently affected in the different brain regions. Corresponding estimates of *V*_T_″ by the linearization of Logan were 10% ± 2% higher than those by compartmental analysis (see Additional file 1). Since this bias was similar in the blocked and unblocked conditions, thus having no significant influence on the calculation of specific binding, the source of the methodology-related differences in estimates of *V*_T_″ has not been investigated further.

The relative changes in *k*_2_″ of [^18^F]NS14490 by NS6740 with the largest effects observed in the hippocampus (−35%) and the lowest reduction measured in the cerebellum (−14%) correlate well (*r* = 0.66) with the density and pattern of α7 nAChR binding sites detected by receptor autoradiography in piglet brain [9]. The BP_ND_ values calculated from the *V*_T_″ values estimated for control and blocking conditions (region-specific between 0.17 and 0.45) are somewhat less than those determined for [^18^F]NS10743 (between 0.24 and 0.76) and [^11^C]NS14492 (between 0.4 and 0.8) in porcine brain [20],[25]. Interestingly, the BP_ND_ values in the same range have been proven suitable to differentiate between healthy controls and patients with Alzheimer's disease with regard to the availability of α4β2 nAChR [40]. The average nonspecific binding, *V*_ND_, of [^18^F]NS14490 in the piglet brain as determined by the occupancy plot (data in Additional file 1) was 2.2 mL cm^−3^ and thus accounts for almost 50% of the *V*_T_″ in regions with high binding site densities, comparable to the ratio of specific-to-nonspecific binding obtained with [^11^C]NS14492 in pig [20] as well as other PET tracers in the human brain, for example 2-[^18^F]FA-85380 [40]. The parametric maps of *V*_T_″ of [^18^F]NS14490 in the control and blocked conditions corroborate the finding of rather diffuse specific binding in the brain (Figure 5). The low apparent displacement may in part be due to the low spatial resolution of the clinically applied but herein preclinically used PET instrumentation, which likely results in considerable loss of signal from small structures in particular in the pig brain. However, the relatively weak displacement effect is largely an inherent problem in the detection of α7 nAChR caused by the comparably low expression of the α7 nAChR protein. Receptor autoradiography in pig brain revealed a maximum density of only 12 pmol g^−1^[9], as compared to 120 pmol g^−1^ for dopamine D_2/3_ receptors in pig caudate [41]. Accordingly, also, the BP_ND_ values calculated from [^18^F]ASEM PET studies in baboons differ only about 40% between low- and high-binding regions [19] which is even less than the difference found in the herein presented PET study (approximately 70%).

In addition to the specific binding of [^18^F]NS14490 in the brain, our data prove an α7 nAChR specific binding component of [^18^F]NS14490 in the large arteries supplying the brain. The BP_ND_ values of 0.18 and 0.11 were calculated in the carotid artery and in the circle of Willis, respectively. This result is consistent with an early observation on α-bungarotoxin binding sites in the carotid body [42], the known presence of α7 nAChR on brain and peripheral endothelial cells [43],[44], the modulation of porcine basilar arteries by presynaptic α7 nAChRs in sympathetic terminals [45] and may, in the context of atherosclerosis, be relevant to an α7 nAChRs- mediated release of inflammatory cytokine by macrophages [46].

Interestingly, the sagittal views of the *V*_T_″ images of [^18^F]ASEM in baboons show extracerebral structures with clear inhibition of *V*_T_″ after the administration of SSR180711, which might be identical to brain vessels [19]. Such result would support our suggestion that α7 nAChR PET radioligands may be suitable to investigate brain vessel diseases in human.

## Conclusions

In conclusion, the present study demonstrated that [^18^F]NS14490 could be used to specifically assess the availability of α7 nAChR in brain structures as well as in the large arteries supplying the brain. An assumed potential of [^18^F]NS14490 to detect α7 nAChR in vulnerable plaques of diseased vessels will be investigated in further studies.

## Abbreviations

BP_ND_: binding potential

*K*_1_: constant of blood-brain clearance

*k*_2_″: apparent washout rate constant from the brain

M: metabolite

MeCN: acetonitrile

nAChR: nicotinic acetylcholine receptor

PET: positron emission tomography

RP: reversed phase

S.D.: standard deviation

SPE: solid-phase extraction

SUV: standardized uptake value

TAC: time-activity curve

VOIs: volumes of interest

*V*_T_″: distribution volume

## Competing interests

Dan Peters, NeuroSearch A/S, is listed as an inventor on patent application WO 2007138037 held by NeuroSearch A/S, covering the preparation and medical use of novel 1,4-diazabicyclo[3.2.2]nonyl oxadiazolyl derivatives including NS10743.

## Authors’ contributions

SR was responsible for the synthesis of the radiotracer and the analysis of radiometabolites. Additionally, he drafted the manuscript concerning the radiochemistry and the section of radiometabolites. WDC was mostly responsible for the biological experiments including the organization and accomplishment of the dynamic PET scans. Additionally, she drafted the manuscript concerning the data obtained from the PET scans. PC analysed the PET data concerning the imaging of α7 nicotinic acetylcholine receptors in the brain. CKD contributed to the experimental accomplishment of the experimental PET scans. MS was responsible for the synthesis of precursor material and assisted in drafting the manuscript. SF assisted in the production of [^18^F]F^−^, the synthesis of the radiotracer, the analysis of radiometabolites and in drafting the manuscript. GX analysed the PET data concerning the potential of [^18^F]NS14490 to be used as radiotracer for imaging cerebral vasculature and assisted in drafting the manuscript. JS contributed to the study concept and revised the manuscript critically. DP contributed to the study concept and assisted in drafting the manuscript. He is the owner of the patent of NS14490 which was used as reference compound and supported the blocking compound NS6740. OS supported the PET studies and revised the manuscript critically. JB analysed and interpreted the PET data concerning the potential of [^18^F]NS14490 to be used as radiotracer for imaging of α7 nicotinic acetylcholine receptors in the cerebral vasculature. PB was responsible for the design of the study, contributed to the experimental accomplishment of the experimental PET scans, performed the statistical analysis and critically revised the manuscript. All authors read and approved the final manuscript.

## Additional file

## Supplementary Material

Additional file 1: **Imaging of α7 nicotinic acetylcholine receptors in brain and cerebral vasculature of juvenile pigs with [**^**18**^**F]NS14490.** The supplemental material provides more detailed information on the radiosynthesis of [^18^F]NS14490 including the experimental setup and the mode of action. The procedure of the *in vitro* autoradiography is described there. It furthermore contains additional information on the results of the *in vitro* autoradiography and the dynamic PET studies in the brain of piglets. Click here for file
