# Supplementary material for: Imaging of α7 nicotinic acetylcholine receptors in brain and cerebral vasculature of juvenile pigs with [18F]NS14490
Source: EJNMMI Res. 2014 Aug 5;4:43. doi: 10.1186/s13550-014-0043-5 (PMC4129469; doi:10.1186/s13550-014-0043-5)
Supplement: Additional file 1: — Imaging of α7 nicotinic acetylcholine receptors in brain and cerebral vasculature of juvenile pigs with [ 18 F]NS14490. The supplemental material provides more detailed information on the radiosynthesis of [18F]NS14490 including the experimental setup and the mode of action. The procedure of the in vitro autoradiography is described there. It furthermore contains additional information on the results of the in vitro autoradiography and the dynamic PET studies in the brain of piglets. [file s13550-014-0043-5-S1.pdf]

Supplemental material to the paper by Rötering et al. entitled:
Imaging of α7 nicotinic acetylcholine receptors in brain and cerebral vasculature of juvenile pigs with [^{18}F]NS14490

Supplement to Material and Methods

Automated synthesis of [^{18}F]NS14490

Automated radiosynthesis of [^{18}F]NS14490 has been developed as follows. Figure 1 presents the set-up of vials and containers of the automatic module, and Table 1 shows which vials were used and how they were filled, and which cartridges were used.

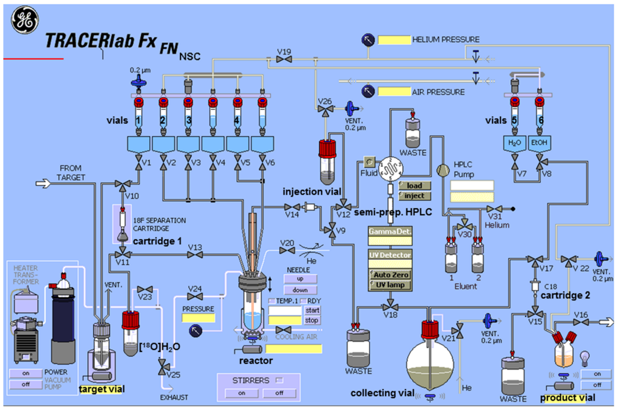

Fig. 1 Configuration of the TRACERlab™ FX F-N automate for the synthesis of [^{18}F]NS14490

An activity of 6.5 - 15.8 GBq n.c.a. [^{18}F]fluoride in aqueous solution placed in the target vial (2 mL, Figure 1), was trapped by passage through an anion exchange cartridge (cartridge 1, Figure 1) on the TRACERlab™ FX-N synthesis module. Activity was released into the reactor upon addition of K_2CO_3 (1.8 mg, 13 mmol) diluted in 400 µL acetonitrile/water 1:1 v/v (vial 1, Figure 1). Next, Kryptofix K_{222} (11.2 mg, 28 mmol) in 1 mL anhydrous acetonitrile (vial 2, Figure 1), was added, and the K[^{18}F]F-K_{222} complex was formed under a flow of helium at 55°C for 6 min. After evaporation at 85°C under vacuum for 2 min, the reactor was cooled to 60°C and the tosylate precursor **1** dissolved in 750 - 800 µL anhydrous acetonitrile (vial 3, Figure 1) was added to the reactor. Nucleophilic substitution was performed by heating to 85°C for 20 min under a helium atmosphere. The reactor was than cooled to 35°C and a sample was taken for radio-TLC. The process was continued by the addition of 3 mL of acetonitrile/water 35%/65% (vial 4, Figure 1) into the reactor and transferring the reaction mixture to the injection vial. After completion of the transfer, the loop was loaded and the mixture injected to the RP-column of the semi-preparative HPLC column. The fraction containing [^{18}F]NS14490 was collected in a vial (Suppl. Figure 1) containing 10 mL water, and the solution was passed through the HR-X cartridge (cartridge 2, Suppl. Figure 1), trapping the radiotracer. The cartridge was washed by 3 mL water (vial 5, Figure 1) and the radiotracer was released into the product vial by passing 1.5 mL acetonitrile/1.5% formic acid (vial 6, Figure 1) through the cartridge. This solution was manually evaporated at 65°C - 70°C and formulated in 200 µL - 400 mL phosphate-buffered saline (PBS) containing 5% ethanol.

In vitro autoradiography of piglet brain

In vitro autoradiography with [^{18}F]NS14490 on sagittal sections (20 µm) of the right brain hemispheres of 3 female piglets was performed based on previously published procedures [1] with slight modifications. The brain slices were incubated for 60 minutes at room temperature in assay buffer (50 mM TRIS-HCl, 120 mM NaCl + 5 mM KCl pH 7.4/21°C) containing 13.7 MBq [^{18}F]NS14490 (0.228 MBq mL^{-1}). Non-specific binding was determined on adjacent sections in the presence of 300 µM (−)-Nicotine hydrogen tartrate salt. Specific binding was calculated as difference between total and non-specific binding. Competition of non-radioactive α7-selective ligands with [^{18}F]NS14490 was investigated with 10 µM of the partial agonist SSR-180,711 and 10 µM of the antagonist methyllycaconitine citrate salt hydrate (MLA). After washing and air-drying all slides were exposed to BAS-SR2325 imaging plates (Fuji Film, Tokyo, Japan) for 24 h. Quantitative analysis of the scan data was performed by computer-assisted microdensitometry. Irregular regions of interest were drawn over selected areas of the brain. The brain regions of interest were confirmed by Nissl and Gallays staining [2] on adjacent sections with the help of a pig brain atlas [3].

Supplemental information to Results

An in vitro autoradiograph of [^{18}F]NS14490 binding on a sagittal sections of piglet brain is shown in Fig.2 (left). Co-incubation with 10 µM of the partial agonist SSR-180,711 resulted in a strong reduction of [^{18}F]NS14490 binding throughout the brain (Fig. 2, rigth). The highest inhibition was found in the hippocampus while the lowest was observed in the cerebellar white matter. Almost identical values were found when 10 µM of the antagonist MLA was added to the incubation solution.

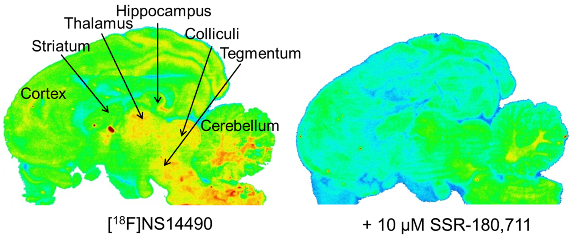

Fig. 2 In vitro autoradiographs of [^{18}F]NS14490 binding on sagittal sections of piglet brain, left: control, right: co-incubation with the α7 nAChR- selective SSR-180,711.

The values of specific binding estimated in the brain regions indicated in Fig. 2 were correlated to the specific binding estimated by PET as difference of V_T under

baseline and blocked condition. A highly significant correlation ($p < 0.002$) was found (Fig. 3).

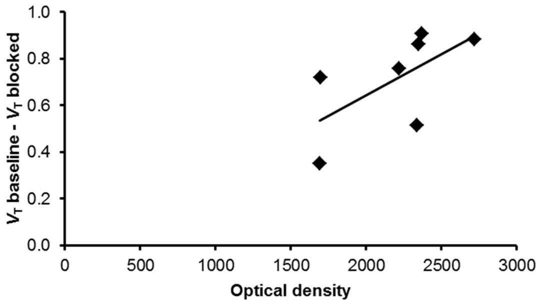

Fig. 3 Correlation between the specific binding of [^{18}F]NS14490 at porcine brain in vitro (optical desnity measured by autoradiography) and the specific binding measured by PET (difference of V_T under baseline and blocked conditions). Data are means of the values measured in various brain regions (cortex, striatum, thalamus, hippocampus, colliculi, midbrain tegmentum, cerebellum), r = 0.63, $p < 0.002$.

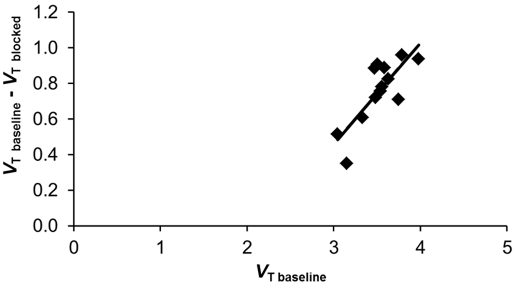

Fig. 4 Occupancy plot of [^{18}F]NS14490 V_Ts in all investigated brain regions. Fraction of α7 nAChR occupancy by the blocking agent NS6740 is calculated as slope of the regression line, and of [^{18}F]NS14490 V_{ND} as intercept at y-axis. r = 0.80, $p < 0.001$.

Furthermore it has been shown that the PET estimate of specific [^{18}F]NS14490 shows strong correlation to V_T under baseline conditions. The respective occupancy plot (Fig. 4) allows to estimate the average nonspecific binding, V_{ND}, of [^{18}F]NS14490 in the piglet brain (2.2 mL cm^{-3}).

Parametric images V_T obtained from Logan Plot represent specific binding of [^{18}F]NS14490 in piglet brain because a strong Correlation between V_T from Logan Plot and V_T from compartmental analysis has been found (Figure 5).

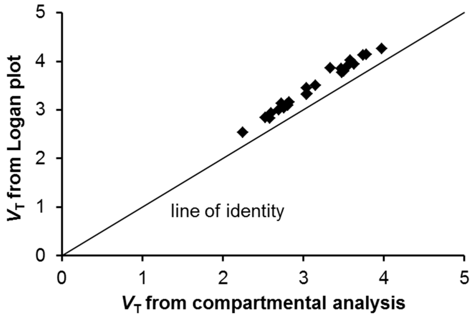

Fig. 5 Correlation between V_T from Logan Plot and V_T from compartmental analysis in all investigated brain regions together with the line of identity, r = 0.99.

REFERENCES

1. Deuther-Conrad W, Wevers A, Becker G, Schildan A, Patt M, Sabri O, Steinbach J, Brust P. Autoradiography of 2-[^{18}F]F-A-85380 on nicotinic acetylcholine receptors in the porcine brain in vitro. Synapse. 2006;59:201-10.
2. Gallyas F. Silver staining of myelin by means of physical development. Neurological research. 1979;1:203-9.
3. Felix B, Leger ME, Albe-Fessard D, Marcilloux JC, Rampin O, Laplace JP. Stereotaxic atlas of the pig brain. Brain Res Bull. 1999;49:1-137.

Table 1 Experimental set-up for the production of [^{18}F]NS14490 in an automated synthesis module. Steps marked with (*) were optimised within the development of the radiosynthesis.

Element	filled/equipped with	volume	Remarks
target vial	[^{18}F]F$^-$ from cyclotron	2 mL, filled with H_2O	
[^{18}O]H_2O	empty		collection of [^{18}O]H_2O for recycling
cartridge 1	PS-HCO_3^- (unconditioned)		separation of activity from [^{18}O]H_2O
reactor	stirrer		
vial 1	1.8 mg K_2CO_3	400 µL water/MeCN 1:1	desorption of the activity into the reactor
vial 2	11.2 mg Kryptofix K_{222}	1 mL MeCN	filled into reactor with activity
vial 3	2 mg tosylate precursor 1	800 µL MeCN	
vial 4	MeCN/water 35:65	4 mL	for transfer to injection vial
injection vial	empty		for transfer to HPLC
semi-prep. HPLC	RP-column*		
collecting vial	water	10 mL	collection of fraction and dilution for SPE
cartridge 2	HR-X (M, 85 µm) (conditioned)		
vial 5	water	3 mL	washing of radiotracer trapped on cartridge
vial 6	desorption agent*	1.5- 3 mL	
product vial	empty		product after SPE for final evaporation and formulation
